# Comparative Evaluation of Synthetic Zeolites for Radium and Barium Removal from Contaminated Water: From Ideal Solutions to Real Mine Water Matrix

**DOI:** 10.3390/ma19112353

**Published:** 2026-06-02

**Authors:** Krzysztof Samolej, Rafał Panek, Damian Stefański, Amin Shahrokhi

**Affiliations:** 1 Silesian Centre for Environmental Radioactivity, Central Mining Institute—National Research Institute, Pl. Gwarków 1, 40-166 Katowice, Poland; dstefanski@gig.eu; 2 Department of Construction Materials Engineering and Geoengineering, Lublin University of Technology, Nadbystrzycka 40, 20-618 Lublin, Poland; r.panek@pollub.pl; 3 Department of Radiochemistry and Radioecology, University of Pannonia, 8200 Veszprem, Hungary; shahrokhi.amin@mk.uni-pannon.hu

**Keywords:** molecular sieves, NORM, ion exchange, adsorption, heavy metals

## Abstract

Radium and barium are hazardous contaminants that frequently occur in wastewater, posing significant risks to human health and the environment. This study provides a comparative evaluation of five synthetic zeolites—3A, 4A, 5A, 13X (commercial), and NaP1 (synthesized from fly ash)—representing three distinct framework types (**LTA**, **FAU**, and **GIS**) for the removal of radium from real saline mine water (Upper Silesia Coal Basin, Poland) and barium from synthetic water. The zeolites were characterized by XRD, SEM-EDS, and N_2_ adsorption, and tested in both granular and fine-powder forms using sequential batch adsorption experiments. For radium removal from mine water, zeolite NaP1 demonstrated superior performance, maintaining low ^226^Ra effluent activity (<1 Bq/L), even after treating ~50 L of water. Zeolites 3A, 4A, 5A, and 13X exhibited significantly lower performance than NaP1, showing poor selectivity for radium. In the barium batch tests, all tested zeolites achieved removal efficiencies exceeding 95% at low initial concentrations (100 mg/L). At higher concentrations (2000 mg/L), zeolites 3A, 4A, and 13X exhibited the highest adsorption capacities, with zeolite 4A achieving the maximum value of approximately 239.9 mg/g. The experiments demonstrated that idealized laboratory conditions can substantially overestimate sorbent performance relative to real water systems.

## 1. Introduction

The alkaline earth metals Be, Mg, Ca, Sr, Ba, and Ra are silvery shining metals with an ns2 electronic configuration [1]. Their electron affinity as well as their ionization energies are low, resulting in the formation of typically divalent cations M^2+^ upon oxidation [1]. Barium and radium are not biodegradable and can accumulate in living organisms, causing various diseases and disorders [2,3]. Their release into the environment as industrial waste constitutes a serious environmental problem that affects human health and ecological systems [4]. It is therefore essential either to remove them from industrial effluents or to reduce their concentrations below permissible limits.

Barium is the 14th most abundant element in the Earth’s crust, with an average crustal abundance of 425 ppm [5]. The most common source of barium is a mineral called barite, which consists of barium sulfate (BaSO_4_) [1].

In contrast, radium occurs in nature only in trace amounts; it has no stable isotopes. The most abundant is ^226^Ra, with half-life of 1600 years and average crustal abundance of 10^−7^ ppm [6].

Barium displays chemical behavior comparable to that of calcium and magnesium; however, its geochemical characteristics align most closely with those of radium. This is primarily due to their similar ionic radii and tendency to connect with sulphate ions. Barium is an ideal analog of radium, as Ba and Ra possess similar ionic radii (Ba^2+^ = 1.34 Å and Ra^2+^ = 1.43 Å) [7]. Consequently, barium and radium frequently co-occur in groundwater environments [8].

Various studies have shown that Ba can cause serious problems for human health. Barium poisoning may cause gastrointestinal, metabolic, cardiovascular, musculoskeletal, and neurological effects [8]. According to the U.S. Environmental Protection Agency (EPA US), exposure to barium may entail: muscular paralysis, stomach irritation, swelling of the brain and liver, and high blood pressure [9].

Both ^226^Ra and ^228^Ra are among the most radiotoxic elements present in the environment [3]. Once radium enters the human body, it can be incorporated into bones and teeth by substituting for calcium; moreover, it is neither metabolized nor efficiently excreted, so its decrease in the body occurs entirely through radioactive decay [10]. The primary health risks associated with radium arise from its radiological properties. Radium exposure can lead to several disorders and immunological effects, including acute leukopenia, with an almost complete absence of granular leukocytes, leukoblastic groups, and lymphoid tissue in the bone marrow, and head carcinomas [11]. An additional hazard, beyond the direct absorption of radium, is posed by its decay products, particularly radon and its short-lived progeny, which attach to airborne aerosols, forming radioactive particles capable of penetrating the lungs [12].

Several methods have been developed and studied to remove barium from water, including: chemical precipitation [13]; flocculation and coagulation [14]; photocatalytic processes [15]; electrodialysis [16]; reverse osmosis [17]; membrane separation [18,19]; and ion exchange by resins [20,21], zeolites [22,23,24,25,26] and nanoparticles [27].

The removal of radium from water can be achieved using a co-precipitation method in the presence of barium, if the water naturally contains barium ions, by adding a material that supplies sulphate ions, whereas in water lacking barium ions but containing sulphate ions, the process can be facilitated by the addition of barium chloride [28]. Other available treatment methods include: filtration [29,30]; reverse osmosis [31]; soda softening technology [32]; and application of adsorbents, such as ion exchange resins [33,34], zeolites [35,36,37,38,39,40,41,42], manganese oxide [43,44,45], and activated carbon [46].

As seen from the literature review, zeolites can be used for removal of Ra and Ba from wastewater. A zeolite is a crystalline aluminosilicate with a 3D framework structure that forms uniformly sized pores of molecular dimension. Tetrahedrally connected frameworks of SiO_4_ and AlO_4_ units generate uniform channels and cages. Isomorphous substitution of Si^4+^ by Al^3+^ in the framework generates a net negative charge, which must be balanced by the cations located within the pore system. Loosely held cations that sit within the cavities preserve the electroneutrality of the zeolite. The International Zeolite Association (IZA) has recognized over 250 unique zeolite framework structures to date 2026 [47].

Zeolites 3A, 4A, and 5A have the same basic **Linde Type A** (**LTA**) crystal structure. The general base formula for zeolite A is [Al_12_Si_12_O_48_]_8_—**LTA** [47]. The framework consists of sodalite cages (β-cage) connected to each other through their four-membered oxygen rings via double four-ring (D4R) units. The interstices within a cubic array of sodalite cages form a simple cubic array of much larger supercages (α-cage) that interconnect by sharing rings of eight tetrahedra. These eight-membered rings are the apertures that define the effective pore size of the zeolite and give the **LTA** zeolites their distinct numbers: 3A, 4A, and 5A. The Na^+^ form of the **LTA** structure has an effective pore size of 4 Å. By exchanging Na^+^ with larger K^+^ ions, these larger ions more significantly block the eight-member apertures, reducing the effective pore size to 3 Å. By exchanging Na^+^ with Ca^2+^ ions, different electrostatic charge densities modify the accessibility of the α-cage, leading to a larger effective pore size of 5 Å.

Zeolite 13X has a **Faujasite** (**FAU**) crystal structure. The general base formula for zeolite 13X is Al_58_Si_134_O_384_—**FAU** [47]. The framework comprises sodalite cages (β-cage) arranged in a diamond-type lattice, connected to each other through their double six-rings. This creates a supercage with four tetrahedrally oriented, 12-ring pore openings, with diameter of about 13 Å. The effective pore opening of this zeolite is a 7.4 Å window, formed by a 12-membered ring, which is the connection point of the supercages.

Zeolite NaP1 has a **Gismondine** (**GIS**) crystal structure. The general base formula for zeolite NaP1 is Al_8_Si_8_O_32_—**GIS** [47]. The framework can be described as a stacking of two-dimensional arrays of double crankshaft chains. There are eight-ring channels running parallel that are cross-linked to form a three-dimensional lattice rather than cages. The entrances to the internal part are defined by the abovementioned eight-rings. The effective pore opening is 3.1 Å.

Synthetic zeolite materials can be obtained not only from pure chemical reagents but also from various waste materials, particularly fly ash (carbon combustion products). Its conventional synthesis utilizes chemical sources of silicon (e.g., sodium waterglass, colloidal silica sol, fumed silica, tetramethylorthosilicate, tetraethylorthosilicate) and aluminum (e.g., sodium aluminate, pseudo-boehmite, aluminum hydroxide, aluminum isopropoxide, aluminum nitrate, aluminum sulfate). However, an alternative and sustainable approach involves the hydrothermal conversion of mineral raw materials (clay minerals, silica minerals) or industrial wastes, such as fly ash. This method typically yields products containing not only zeolite phases but also an unreacted residuum composed of aluminosilicate glass, mullite, and quartz [48], and they may form mixtures of two or more zeolite types rather than a single monomineralic phase [49]. Panek et al. [50] demonstrated that ultrapure zeolite materials can be synthesized from fly ash, utilizing the waste solution generated during the initial synthesis as the raw substrate for producing high-purity zeolitic products.

In this study, radium removal from real saline mine water and barium removal from synthetic water were evaluated using five synthetic zeolites: 3A, 4A, 5A, 13X, and NaP1.

## 2. Materials and Methods

### 2.1. Zeolites and Water

**Figure 1:**
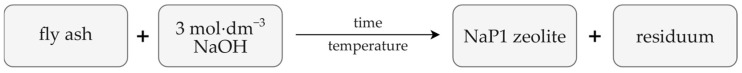
The synthesis reaction of NaP1 according to Franus et al. [51].

Zeolites 3A, 4A, 5A, 13X and NaP1 were selected for the experiments. The fly ash-derived zeolitic material NaP1 was produced by hydrothermal synthesis from fly ash. Using an F-class ash from the combustion of coal and sodium hydroxide, hydrothermal conversion was performed according to the procedure presented by Franus et al. [51]. The synthesis of the NaP1 phase was performed according to the chemical reaction presented in Figure 1, using the following parameters: 20 g of fly ash mixed with 0.5 dm^3^ of 3 mol·dm^−3^ NaOH solution for 24 h at 95 °C [51]. We acknowledge that the synthesis methodology for NaP1 presented above was also used to produce the sample of zeolites examined and tested in our earlier work [52]. The present study, however, used a different industrial batch of fly ash, zeolite was prepared as a separate production batch, and focused on comparative evaluation of NaP1, 3A, 4A, 5A, 13X for removal of radium and barium from different solutions. All the characterization data (XRD, XRF, SEM-EDS, N_2_ adsorption/desorption) reported herein were independently acquired for this specific batch using the equipment and protocols detailed here in the Materials and Methods section, and are therefore original results. The present work extends beyond our previous study by providing a comparative evaluation of zeolites representing **GIS**, **LTA** and **FAU** frameworks under directly comparable conditions for radium removal from real saline mine water and barium removal from synthetic solutions.

Zeolites 3A, 4A, 5A and 13X are commercial products and were purchased from XINTAO (Guangdong Xintao Technology Co., Ltd., Guangzhou, China). The characterization of zeolites was performed using X-ray diffraction, scanning electron microscopy (SEM), and the volumetric gas sorption method. The zeolites selected for the experiments were in two different forms: spherical granules with diameters 1.7–2.4 mm—3A, 4A, 5A, and 13X—and fine powders with a size 0.125–0.180 mm—NaP1, 3A, 4A, 5A, and 13X. The technical characteristics of the granulated zeolite samples are presented in Table 1. Fine-powder form of zeolites 3A, 4A, 5A and 13X was prepared by crushing granules and separating the desired fraction by a sieve analysis. The NaP1 was in fine-powder form after its synthesis; a sieve analysis was used to isolate the desired fraction.

XRD was used for the phase composition analysis. It was determined based on the X-ray diffraction spectra (2θ from 5 to 70°) obtained in a diffractometer (D8 DISCOVER, Bruker, Karlsruhe, Germany) equipped with a CuKα radiation source and a LUNXEYE_XE Ni filter detector (Bruker, Karlsruhe, Germany). The phase identification was performed using the International Centre for Diffraction Data (ICDD) PDF-4+ 2019 RDB [53] and Inorganic Crystal Structure Database (ICSD) [54]. The quantitative analysis was carried out using the Rietveld refinement method [55].

Morphology of zeolites was observed using a scanning electron microscope (Quanta 250 FEG, manufactured by FEI, Hillsboro, OR, USA) in conjunction with an EDAX-produced EDS chemical composition analysis tool. The images were captured at 16,000× magnification under an accelerating voltage of 10 kV with a BSD detector. Elemental analysis of zeolites was performed using an energy-dispersive X-ray spectrometer (EDS). To obtain high-quality images, the samples were coated with a gold layer using a sputtering device (CCU-010 LV, Safematic GmbH, Zizers, Switzerland).

The textural characteristics of the studied samples were determined using a 3Flex 3500 surface area and porosity analyzer (Micromeritics Instrument Corp., Norcross, GA, USA). Zeolites 3A, 4A, 5A and 13X were examined in the form of granules, while NaP1 was examined in powder form. The nitrogen adsorption–desorption measurements were performed at 77 K (−194.85 °C) within a relative pressure (p/p_0_) range of 1.5 × 10^−7^—0.90. Prior to the measurements, the materials were degassed under reduced pressure at 250 °C for 24 h using the instrument’s degassing station to remove the physically adsorbed species and obtain a clean sample surface. The BET specific surface area (S_BET_) and the Langmuir specific surface area (S_Langmuir_) were calculated from the adsorption branch of the N_2_ isotherm at 77 K [56,57]. The micropore surface area (S_mic_), external specific surface area (S_ext_), and micropore volume (V_mic_) are apparent values derived from t-plot analysis using the Harkins–Jura thickness equation [58,59]. The total pore volume (V_total_) was calculated from the amount adsorbed at p/p_0_ = 0.90 [60].

Synthetic water containing barium was prepared using barium chloride dihydrate (BaCl_2_·2H_2_O), supplied by the Polish manufacturer Polskie Odczynniki Chemiczne (POCH, Gliwice, Poland), and deionized water. The prepared concentrations were in the range of 100–2000 mg/L. A ^133^Ba tracer, supplied by the National Centre for Nuclear Research Radioisotope Centre (POLATOM, Otwock, Poland), was used to determine the barium concentrations in water, and the β particles were counted using liquid scintillation spectroscopy (LSC) (QUANTULUS model 1220, Wallac, Turku, Finland).

The water with radium content was collected from a surface sedimentation pond for mine water belonging to a hard coal mine located in the Upper Silesia Coal Basin (USCB) in Silesian Voivodeship in Poland. It is a radium sulphate type of water, containing radium and sulphates as well as trace amounts of barium ions [61]. The water in the sedimentation pond is a mixture of water pumped from the underground mine workings and surface water/rainwater.

Concentrations of radium (^226^Ra and ^228^Ra) in the water were determined by liquid scintillation spectroscopy (QUANTULUS model 1220, Wallac, Turku, Finland), after chemical separation of radium as Ba(Ra)SO_4_ within the barium carrier, using an INSTA-GEL^®^ PLUS gel scintillator (Packard Instrument Co., Meriden, CT, USA) [62].

The concentration of the elements present in the mine water samples was determined by inductively coupled plasma optical emission spectrometry (ICP-OES) (Optima model 5300DV, PerkinElmer Inc., Shelton, CT, USA).

### 2.2. Batch Adsorption Studies

Sorption studies of radium on zeolites were conducted using the sequential batch method. The batch adsorption experiments were conducted using 5 g of adsorbent and 1 L of mine water containing radium (pH = 7.2). The water and zeolite were placed in a 2 L glass beaker and mixed for 3 h using a magnetic stirrer. After mixing, the sample was set aside for 1 h to allow the suspension to settle. The total time the zeolite remained in contact with the water in one sequence was 4 h. After treatment, the water sample was filtered through a 0.45 µm filter. This procedure was repeated for successive water samples in the same sequence for the same zeolite. The radioactive radium concentration measurement was carried out by LSC. The elemental content measurement was performed by ICP-OES on the water samples before treatment.

Sorption studies of barium on zeolites were conducted using the batch method. The experiments were performed in polyethylene bottles, to which were added 5 g of zeolite and 1 L of solution containing appropriate barium ions concentrations and ^133^Ba tracer (pH = 6.8). The bottles were agitated every 24 h. The contact time to establish equilibrium between the zeolite and solution was 60 days; the temperature of the water batch was maintained at 21 °C. After equilibration, the water was separated by filtration through a 0.45 µm filter. The barium concentration measurement was carried out by LSC.

The removal efficiency (η) was calculated using the following equation:

(1) $$\eta =\frac{C_{i}-C_{f}}{C_{i}}\times 100\%,$$

where C_i_ (Bq/L) and C_f_ (Bq/L) are the concentrations of the impurity in the water in the initial and final solutions, respectively.

The equilibrium adsorption capacity (q_e_) was calculated using the following equation:

(2) $$q_{e}=\;\frac{\left(C_{0}-C_{e}\right)V}{m},$$

where C_0_—initial concentration of impurity in the solution (mg/L); C_e_—equilibrium concentration of impurity in the solution (mg/L); V—volume of solution (L); and m—zeolite mass (g).

## 3. Results and Discussion

### 3.1. Properties of the Water

The collected mine water contained both radium isotopes with the following radium concentrations: ^226^Ra 2.2 ± 0.2 Bq/L and ^228^Ra 3.9 ± 0.5 Bq/L. The water sample in question was of the radium sulphate type, with a trace amount of barium of 0.2 ± 0.02 mg/L and a sulphate content of 2800 ± 300 mg/L. The collected water sample exhibited a very high electrical conductivity of 100,000 µS/cm and the total dissolved solids were 34.0 ± 3.9 g/L. The results of the water chemical analysis are presented in Table 2.

### 3.2. Properties of the Zeolites

**Figure 2:**
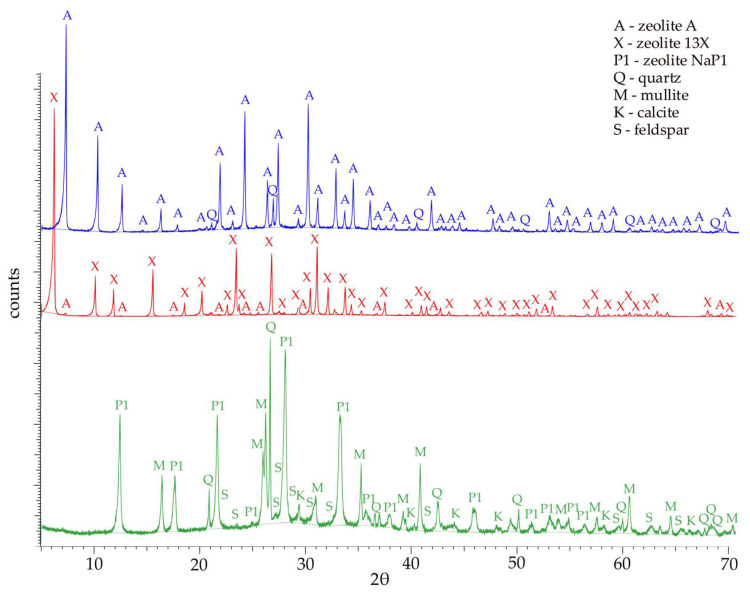
XRD diffraction patterns of zeolites: NaP1, 3A, 4A, 5A and 13X.

The mineralogical composition of the zeolites was determined by XRD, and the diffraction patterns are presented in Figure 2. The zeolite phase content was evidenced by the main d-spacing d_hkl_: 7.101 Å, 4.100 Å, and 3.176 Å for NaP1 (card no. PDF 039-0219); 14.470 Å, 3.808 Å, and 2.885 Å for 13X (card no. PDF 038-0237); and 12.304 Å, 8.701 Å, and 2.987 Å (PDF 039-0222) for 3A, 4A, 4A and 5A. The diffractometric analysis confirmed the presence of zeolite phases as declared by the manufacturers and enabled the division of the zeolites into groups according to their framework types: **GIS**—NaP1, **FAU**—13X, and **LTA**—3A, 4A, and 5A; the zeolite phase contents found in the samples were 48%, 84%, 77%, 80% and 84%, respectively. The diffraction patterns for all studied samples showed the presence of crystalline impurities or an amorphous phase.

The characteristic increase in the background of the XRD pattern was likely caused by an aluminosilicate glass formed from aluminum and silicon oxides, with an amorphous non-crystalline phase [63,64]. The most diverse mineral composition was observed in the NaP1 sample, which contained 48% zeolite phase, 13.5% quartz, 12% mullite, 7.0% feldspar, 1.5% calcite, and 18% amorphous phase. The presence of quartz, mullite, feldspar, and calcite resulted from the raw material (fly ash) used for zeolite synthesis [51]. The major components of raw fly ash are silica (SiO_2_) and alumina (Al_2_O_3_). During alkaline activation, these are converted to zeolite; however, a portion remains unreacted, while another is transformed into aluminosilicate glass, which is visible as the elevated background [65,66].

In samples 3A, 4A, and 5A, only trace amounts of quartz were detected in addition to the zeolite phase (3.5%, 2.0%, and 1.5%, respectively). The amorphous phase mentioned earlier was also present at concentrations of 19.5%, 18.0%, and 14.5% in samples 3A, 4A, and 5A, respectively. Both components were residues of unreacted materials used in zeolite synthesis [67,68].

In the 13X sample, in addition to the primary X zeolite phase, trace amounts of LTA zeolite (1.5%) were identified. The presence of this minor LTA phase is associated with the synthesis process, during which competing crystalline phases can form. This occurs because different zeolite structures exhibit similar stability under comparable conditions, combined with the imperfect mixing and heterogeneity of the system [69,70]. The remaining amorphous phase, which could not be identified due to its non-crystalline nature, was a residue from the incomplete zeolite crystallization and unreacted raw materials [67].

**Figure 3:**
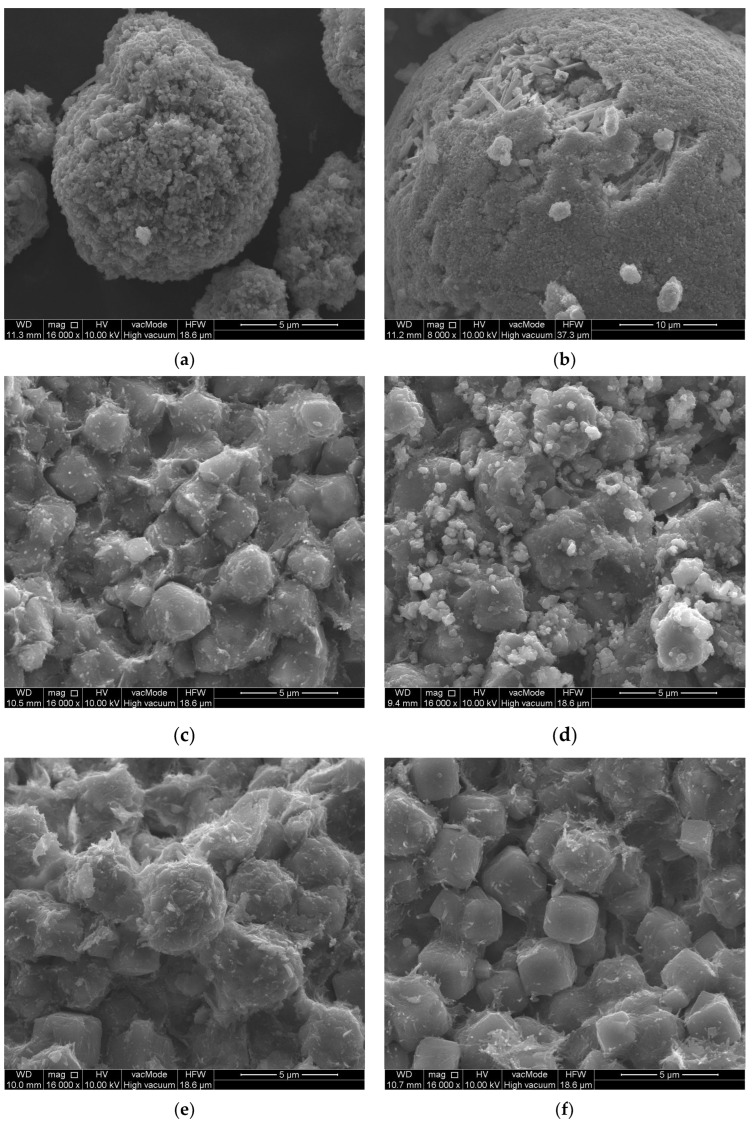
SEM images of zeolites: (**a**) NaP1; (**b**) NaP1; (**c**) 13X; (**d**) 3A; (**e**) 4A; (**f**) 5A.

Surface imaging of the zeolites was conducted under conventional high-vacuum conditions typical for the characterization of microporous materials. The imaging parameters were set to a magnification of 16,000×/8000× with a horizontal field width (HFW) of 18.6 μm/37.3 μm (corresponding to a 5 μm/10 μm scale bar), ensuring a suitable compromise between detailed surface resolution and the broader morphological context of the aggregates. Figure 3 shows microphotographs of the surfaces of individual zeolites.

Regarding the morphological and phase compositions of the NaP1 sample, they indicate a composite material consisting of 48% crystalline zeolite NaP1 and residual unreacted phases originating from the precursor coal fly ash. The SEM image in Figure 3a reveals that well-developed zeolite NaP1 crystallized directly on the surface of these glassy aluminosilicate microspheres as cenospheres. Cenospheres, which are lightweight, hollow microspheres produced during coal combustion, serve as the reactive substrate for zeolite growth. The SEM micrograph of NaP1, in Figure 3a, reveals quasi-spherical secondary particles, with spherulites as the dominant morphological feature. The primary microsphere exhibits a diameter of approximately 10 μm, whereas several smaller microspheres (2–5 µm) are also present in the field of view, partially extending beyond the image frame. These microspheres display a characteristic “cauliflower-like” morphology, indicating that they constitute polycrystalline aggregates rather than single crystals. Furthermore, the surfaces of these microspheres are highly irregular and corrugated, characterized by numerous intercrystalline cracks and cavities, indicating a high external surface area that significantly exceeds that of smooth, single-crystal zeolites. Such a structure is typical for zeolites synthesized via hydrothermal methods, wherein small primary crystals aggregate to form larger secondary particles. In contrast, the remaining 52% of the material consists of unreacted phases, primarily quartz, amorphous glass, and mullite. As shown in Figure 3b, one of them, not fully converted into zeolite mullite, typically occurs as a characteristic needle-like (acicular) crystal.

For zeolite 13X, in Figure 3c, the SEM micrograph reveals that the material is composed of strongly agglomerated, isometric crystals ranging 0.5–2.5 μm in size. These crystals form polycrystalline aggregates with a mosaic-like texture. The grains exhibit irregular, partially rounded edges and rough, defect-rich surfaces, indicating the presence of amorphous overgrowths and surface etching. Additionally, the edges of some grains appear locally fused with neighboring grains, which may be a consequence of the granulation process.

The SEM image of zeolite 3A, in Figure 3d, shows that the material is composed of densely packed zeolite crystals in the range of 0.5–2.5 μm, forming polycrystalline agglomerates with intercrystallite macropores. The external surfaces of these are densely decorated with a secondary population of intergrown nanocrystallites, resulting in a rugged raspberry-like surface architecture. The micrograph shows that the zeolite crystals and their secondary decorations form compact agglomerates, with many grains seemingly in close contact or partially fused at their edges. Such consolidation is typical of industrially relevant zeolite forms, such as granules and pellets.

The SEM image of zeolite 4A, in Figure 3e, is filled with densely agglomerated zeolite particles that are strongly intergrown, typical of granule-forming zeolites. The most distinguishable particles have dimensions ranging 2–5 μm in size. These agglomerates exhibit a quasi-spheroidal morphology, constructed from intergrown primary crystallites. Their surfaces exhibit a distinct plate-like appearance, which is the most visible in all the presented images of zeolites.

The image of 5A zeolite, in Figure 3f, reveals a population of well-defined, faceted crystallites exhibiting a dominant cubic form, with several crystals exhibiting slightly truncated corners. Edge lengths of crystals range from approximately 1.5 to 3.0 µm. The high contrast between the bright edge facets and darker face centers confirms the high degree of crystallinity.

The microchemical analyses were performed using EDS. The elemental compositions were expressed as atomic percentages (At%), normalized to an oxygen-free basis, and used to determine the relative molar ratios of the Si:Al framework and exchangeable cations. The results of the analyses are presented in Table 3.

The microchemical analyses show that the main exchangeable cation for 4A, 13X, and NaP1 is sodium, confirming that they are sodium-type zeolites. Zeolite 3A is a potassium-type zeolite, and its main exchangeable cation is potassium (19.1 At% in the fine-powder form), although sodium is also present (6.9 At%). Similarly, zeolite 5A is a calcium-type zeolite with calcium as its primary exchangeable cation (11.0 At% in the fine-powder form), alongside a residual sodium content (6.9 At%). The analysis of the Si:Al ratio indicates that all the investigated materials can be classified as low-silica zeolites.

The noticeable differences in the chemical composition between the intact granules and the fine powder obtained from crushing them were primarily due to the binder used during the granulation process. Typically, 15% to 25% of an inorganic binder, most commonly clay minerals, such as kaolin, bentonite, or attapulgite, is added to a pure crystalline zeolite powder [71]. The higher Si:Al ratio observed on the surface of the granules compared to the fine powder indicates the presence of a silica-rich clay binder, which tends to concentrate on the outer surface of a pellet during extrusion. This phenomenon was evident for all the tested granulated zeolites, with the Si:Al ratio consistently higher for the intact granules.

Furthermore, the elevated magnesium content detected on the granule surfaces suggests that the magnesium originated from the binder. The specific binder used by the manufacturer may have been attapulgite, a magnesium aluminum phyllosilicate widely used in commercial zeolite granulation [72].

Differences between the fine powder and the intact granules were also observed regarding the exchangeable cations. The concentration of the primary exchangeable ions (Na, K, Ca) was systematically higher in the fine-powder form. For instance, in zeolite 3A, the primary potassium cation constituted 19.1 At% in the fine powder versus 15.9 At% on the granule surface. Similarly, for zeolite 4A, the sodium levels were 21.0 At% (fine powder) compared to 18.8 At% (granules). Crushing the granules exposed their bulk interior, which consisted predominantly of a highly crystalline zeolite phase. This interior contained a higher density of exchangeable cations required to balance the negative framework charge, unlike the binder-rich surface layer, which possessed a much lower cation exchange capacity.

The textural parameters of zeolites 3A, 4A, 5A, NaP1, and 13X, determined by the nitrogen adsorption/desorption method, are presented in a Table 4. The S_BET_ was calculated from the adsorption branch of the N_2_ isotherm using the Brunauer–Emmett–Teller (BET) equation [56]. The linear range for the BET fit was selected individually for each sample because the studied zeolites differed significantly in their pore accessibility and adsorption mechanisms [58]. The chosen relative pressure ranges corresponded to the linear portion of the BET plot and were verified according to consistency criteria, which included a positive value of the BET constant C and a high linear correlation coefficient [58]. The molecular cross-sectional area of the N_2_ molecule was assumed to be 0.162 nm^2^. The relative pressure ranges used for the BET fitting were as follows: 0.1601–0.3696 for zeolite 3A, 0.1360–0.3114 for zeolite 4A, 0.000843–0.03486 for zeolite 5A, 0.03484–0.31089 for zeolite NaP1, and 0.000852–0.03412 for zeolite 13X. The corresponding BET C constant values were 7.96, 33.95, 5898.56, 143.10, and 21,856.17, respectively. The S_Langmuir_ was calculated using the Langmuir equation and was reported as a comparative parameter [57]. This is particularly useful for strongly microporous zeolites, where the classical assumptions of the BET method may not be fully satisfied. The S_mic_, S_ext_, and V_mic_ were determined by the t-plot method using the Harkins–Jura equation for statistical layer thickness [59]. The S_ext_ was calculated from the slope of the selected linear segment of the t-plot, while the micropore volume was obtained from the intercept with the ordinate axis. The S_mic_ area was reported only when the t-plot analysis yielded physically meaningful results [58]. For zeolites 3A and 4A, the t-plot analysis produced a negative V_mic_ and an S_ext_ higher than the S_BET_; therefore, the values of S_mic_ and V_mic_ were not reported for these samples. The obtained textural parameters should be treated as apparent values, reflecting the limited accessibility of the LTA micropore system to N_2_ molecules at 77 K.

Zeolite 13X exhibits a clearly microporous character, which is confirmed by both the nitrogen adsorption–desorption isotherm and the results of the textural analysis. The shape of the isotherm corresponds to a Type I isotherm, typical for microporous materials, in which adsorption occurs primarily through micropore filling. The S_BET_ is 631.9 m^2^/g, whereas S_Langmuir_ reaches 724.1 m^2^/g. The shape of the isotherm suggests that the adsorption description for this material is closer to the Langmuir model than to the classical BET approach. This observation is consistent with the nature of zeolites, for which BET assumptions are not always fully satisfied.

In the case of 13X zeolites, the interpretation of nitrogen adsorption results requires particular caution. Nitrogen is not an ideal adsorbate for the characterization of narrow zeolite micropores, due to, among other things, diffusion limitations and specific interactions. This is reflected by the very high value of the BET constant C (21,856), which indicates very strong adsorbate–adsorbent interactions and further suggests that the material is better described by the Langmuir model than by BET. Although the linear correlation coefficient for the BET plot is very high (R = 0.9999983), this does not automatically imply the physical validity of the BET model for the investigated material. Therefore, the BET surface area in this case should be treated as a comparative parameter rather than an absolutely reliable description of the surface development.

A more informative description is provided by the t-plot analysis, calculated using the Harkins–Jura equation, which is frequently used for oxide materials, and recognized as one of the more appropriate reference bases for interpreting zeolite data [59]. According to this method, the S_mic_ is 676.8 m^2^/g, whereas the S_ext_ is only 47.3 m^2^/g. These results clearly indicate that the major contribution to the total surface area originates from the micropores, while the contribution of the external surface area is minor. The V_mic_ determined by the t-plot method is 0.2203 cm^3^/g, which further confirms the dominance of microporosity in the sample structure.

The pore size distribution analysis using the Horvath–Kawazoe (HK) method indicates a characteristic pore width of approximately 0.525 nm, which corresponds to the typical range of micropores present in 13X zeolite structures. This is confirmed by the DFT analysis, which confirms the presence of a dominant pore fraction in the sub-nanometer range, with the main distribution peak appearing at a width of approximately 0.736 nm. The difference between the HK and DFT analyses results from the differing theoretical assumptions of both methods and the different adsorption interaction models employed during the calculations. Nevertheless, both analyses are consistent and indicate the presence of very narrow micropores characteristic of 13X synthetic zeolites. The V_total_ determined at p/p_0_ = 0.90 is 0.2730 cm^3^/g. Combined with the high micropore surface area and the Type I isotherm, these results confirm that 13X is a material with a highly developed well-ordered microporous structure [58].

Zeolite 5A also exhibits a clearly microporous character, with a window diameter of approximately 0.5 nm (5 Å). Based on the nitrogen adsorption–desorption isotherm at 77 K, the material is classified as exhibiting a Type I(a) isotherm, according to the IUPAC recommendations, which is typical for solids containing primarily narrow micropores with widths below ~1 nm [58]. The S_BET_ is 522.7 m^2^/g, while the S_Langmuir_ calculated from the Langmuir equation reaches 630.8 m^2^/g. Analogous to zeolite 13X, the higher Langmuir surface area suggests that the description of the adsorption process is closer to a monolayer model than to the classical BET approach. This is further confirmed by the high value of the C constant (5898), indicating very strong adsorbate–adsorbent interactions within the micropores. Although this value is lower than that of zeolite 13X, it still exceeds the range considered characteristic of a physically reliable BET interpretation. Similarly, the linear correlation coefficient (R = 0.9999932) does not determine the physical validity of the model but merely reflects the high quality of the mathematical fit. The BET surface area for zeolite 5A, as with other highly microporous zeolites, should be treated primarily as a comparative parameter.

More adequate information regarding the surface is provided by the t-plot analysis, calculated using the Harkins–Jura equation [59]. The S_mic_ is 420.3 m^2^/g, while the S_ext_ reaches 102.4 m^2^/g, with a V_mic_ of 0.1619 cm^3^/g. Compared to zeolite 13X (S_mic_ = 676.8 m^2^/g, S_ext_ = 47.31 m^2^/g), zeolite 5A exhibits a lower proportion of micropore surface area, but simultaneously a significantly larger external surface area. Such a distribution may result from smaller crystallite sizes, greater heterogeneity of the adsorbent grains, or the presence of additional mesoporosity in the material. However, the microporous contribution remains dominant, at approximately 80% of the total surface area, which clearly confirms the strongly microporous structure.

The pore size distribution analysis using the Horvath–Kawazoe (HK) method indicates a median pore width of 0.5235 nm, which is close to the value obtained for zeolite 13X (0.5253 nm) and corresponds to the range of narrow micropores typical for zeolites with sodalite cage structures. A sharp increase in the cumulative pore volume occurs in the 0.47–0.60 nm range, confirming the dominant role of a uniform fraction of narrow micropores. These results are corroborated by the DFT analysis (N_2_ model, cylindrical pores, oxide surface), in which the main pore volume distribution peak occurs at a width of 0.736 nm. The discrepancy between the HK and DFT results regarding the location of the maximum results from the different physical assumptions of the two procedures.

Unlike zeolite 13X, the BJH analysis of the desorption branch for zeolite 5A indicates a non-zero mesopore contribution. The cumulative pore surface area in the mesopore range is 83.9 m^2^/g, and the volume is 0.165 cm^3^/g. The average pore width calculated from the 4V/A relation is 9.23 nm, whereas the peak in the pore size distribution is located at 3.19 nm. The mesoporosity may have resulted from the binder used in the zeolite granulation process. The V_total_, determined by the single-point method at p/p_0_ = 0.90, is 0.2619 cm^3^/g. Collectively, the high micropore surface area from the t-plot (420.3 m^2^/g), the dominant sub-nanometer DFT peak, and the Type I(a) isotherm confirm that sample 5A possesses a highly developed microporous structure with a secondary mesoporous component [58].

NaP1, synthesized from fly ash, exhibits a textural character distinct from that of strongly microporous X-type zeolites. Based on the shape of the nitrogen adsorption–desorption isotherm, the material can be classified as a system with the dominant contribution of mesopores, with an isotherm corresponding to Type IV(a), according to the IUPAC classification [58]. This type of isotherm is characteristic of materials in which, in addition to multilayer adsorption, capillary condensation in the mesopores also plays an important role.

S_BET_ of the investigated sample is 50.1 m^2^/g, whereas the S_Langmuir_ reaches 68.1 m^2^/g. In contrast to strongly microporous zeolites, the application of the BET model appears to be more justified for this material, as confirmed by the moderate value of the BET constant (C = 143.10) and the very good linear correlation coefficient (R = 0.9999507).

The t-plot analysis, calculated with the BET equation, indicates the very small contribution of micropores to the structure of the investigated material. The S_mic_ is only 1.121 m^2^/g, with a corresponding V_mic_ of 0.0013 cm^3^/g, whereas the S_ext_ reaches 49.0 m^2^/g. This means that practically the entire surface development of the investigated adsorbent is associated with the external surface and with a pore system larger than that of classical micropores. These results confirm that NaP1 synthesized from fly ash is not a material with dominant microporosity, but rather a mesoporous or micro-mesoporous system with the clear predominance of the mesoporous component. Such a structural character is consistent with the literature reports for NaP1 zeolites synthesized from fly ash, in which the texture is often co-shaped by the residual aluminosilicate phase.

Additional information on the microporous component is provided by the Horvath–Kawazoe method, for which the median pore width is 0.8231 nm. However, it should be emphasized that, at such a low micropore volume, the HK result is only of auxiliary significance and should not be treated as the primary description of the structure. The presence of a signal in the region of approximately 0.8 nm may be considered an indication of a small fraction of narrow pores. However, its quantitative contribution remains marginal relative to the overall texture of the sample and may rather correspond to the intercrystallite porosity between the zeolite crystallites.

The DFT analysis further confirms the mesoporous character of the sample, although the location of the pore distribution maximum differs from the BJH result, which is a natural consequence of the different physical assumptions of the two methods. The greatest increase in pore volume in the DFT model is observed in the range of approximately 12–15 nm (maximum at approximately 14.76 nm), while the V_total_ determined by this method is 0.21904 cm^3^/g. The discrepancy between the BJH and DFT results stems from the use of different interpretive models and the varying sensitivity of these methods to the pore geometry and distribution. In practice, both approaches lead to the consistent conclusion that the pore structure of sample NaP1 is dominated by mesopores, with a minimal contribution of classic microporosity.

Sample 4A represents a different case from the perspective of the methodological limitations of low-temperature nitrogen adsorption. Zeolite 4A is characterized by an effective pore window diameter of only approximately 0.4 nm (4 Å), which is close to the kinetic diameter of the nitrogen molecule (~0.364 nm). Consequently, the diffusion of N_2_ into the microporous structure is strongly restricted at 77 K, and a substantial part of the internal surface of the sodalite cages remains inaccessible to the adsorbate. This fundamental diffusion barrier affects all the textural parameters determined on the basis of this measurement.

The S_BET_ is only 64.9 m^2^/g, which is significantly lower than the values obtained for the 5A zeolite (522.7 m^2^/g) and 13X zeolite (631.9 m^2^/g), despite the fact that all three materials possess a related internal cage structure with a highly developed internal surface. Such a low BET value does not reflect the true internal surface area of 4A zeolite, but is instead a consequence of the abovementioned inaccessibility of the micropores to N_2_ molecules. It should therefore be assumed that the measured surface area corresponds predominantly to the external surface of the crystallites, and to only a fragmentary, diffusion-limited penetration of nitrogen into the pore system. This occurs despite the fact that the C parameter remains within a reasonable range. In particular, the ratio of S_Langmuir_ (272.6 m^2^/g) to S_BET_ (64.9 m^2^/g), equal to 4.2:1, reflects a situation in which neither of the classical adsorption models adequately describes the isothermal behavior of a material subject to such severe diffusion limitations. Under conditions of full accessibility of N_2_ to the **LTA** structure—as in the case of 5A zeolite after ion exchange with Ca^2+^—the surface area exceeds 500 m^2^/g; for the 4A zeolite, the nitrogen registers only a fraction of this value.

The t-plot analysis yields a negative result, and the determined S_ext_ (76.9 m^2^/g) exceeds the S_BET_ (64.9 m^2^/g). Under such conditions, the reporting of the micropore surface area is not possible. This result is an unambiguous indication that the standard t-plot procedure is not applicable to the characterization of 4A zeolite using N_2_ as the adsorptive at 77 K. The Horváth–Kawazoe analysis gives a median pore width of 0.5118 nm, but at a very low cumulative pore volume not exceeding 0.0149 cm^3^/g. For comparison, the corresponding value for the 5A zeolite (with accessible pores) is 0.165 cm^3^/g. The HK data for sample 4A indicate a slow, nearly linear increase in the cumulative pore volume over the range of 0.46–1.17 nm, without the distinct step characteristic of efficient micropore filling. This confirms that nitrogen penetrates the structure only to a very limited extent.

The DFT analysis (N_2_ model, cylindrical pores, oxide surface) leads to consistent conclusions. In the DFT pore size distribution, no distinct dominant peak is observed in the sub-nanometer range, in contrast to the 5A and 13X zeolites, for which the main maximums occur at 0.736 nm. This demonstrates that, in this case, the DFT mainly registers the secondary porosity (intercrystalline and mesoporous) rather than filling of the actual micropores of the **LTA** structure. At the same time, BJH analysis of the desorption branch indicates a certain mesopore contribution. The cumulative pore surface area is 66.04 m^2^/g, and the corresponding pore volume is 0.1489 cm^3^/g, with an average pore width of 4V/A = 9.02 nm. The presence of this mesoporosity may be a consequence of the granulation method used for the commercial material, the presence of a binder, or the intercrystalline porosity.

The total pore volume determined by the single-point method at p/p_0_ = 0.90 is only 0.0763 cm^3^/g for the adsorption branch and 0.0839 cm^3^/g for the desorption branch, which represents only a fraction of the values obtained for the 5A zeolite (0.2619 cm^3^/g) and 13X zeolite (0.2730 cm^3^/g). The zeolite sample 4A therefore constitutes an example of a zeolite whose internal microporosity is inaccessible to nitrogen molecules under standard adsorption measurement conditions at 77 K. All the determined textural parameters (BET, t-plot, HK, and DFT) confirm the predominance of diffusion limitations and indicate that the measured values describe primarily the external surface and secondary porosity, composed mainly of mesopores.

Zeolite 3A represents an extreme case of micropore inaccessibility to nitrogen molecules under standard adsorption measurement conditions at 77 K. The effective pore window diameter of 3A zeolite is approximately 0.3 nm (3 Å), and is therefore clearly smaller than the kinetic diameter of a N_2_ molecule (~0.364 nm). As a consequence, nitrogen is probably completely excluded from the internal micropore space, and the recorded adsorption phenomena most likely occur on the external surface of the crystallites and within the secondary (intercrystalline) porosity.

The S_BET_ is only 45.2 m^2^/g, and the value of the BET constant (C = 7.96) is very low, indicating a complete lack of strong adsorbate–micropore interactions. According to the IUPAC recommendations, a C value below 20 indicates the absence of a physically meaningful BET interpretation and is characteristic of materials in which N_2_ interacts only with the external surface, with weak multilayer adsorption [58]. The S_Langmuir_ reaches 289.23 m^2^/g

The t-plot analysis yields results analogous to those obtained for the 4A zeolite. The calculated micropore volume is negative, and the determined S_ext_ (67.0 m^2^/g) again exceeds the total BET surface area (45.2 m^2^/g), which confirms the complete unsuitability of the standard t-plot analysis for a material into which N_2_ does not penetrate [73]. The Horvath–Kawazoe analysis gives a median pore width of 1.372 nm, which already falls within the mesopore range. The shift of the HK median to values above 1 nm indicates that the method is registering secondary porosity rather than true micropores.

The results of the DFT analysis (N_2_ model, cylindrical pores, oxide surface) also do not provide an unambiguous description, and the DFT model shows only limited agreement with the experimental nitrogen adsorption isotherm for the 3A zeolite. BJH analysis of the desorption branch indicates the presence of a mesopore-related contribution, with a cumulative pore surface area of 48.22 m^2^/g and a pore volume of 0.1440 cm^3^/g, with an average pore width of 4V/A = 11.94 nm. These parameters are close to the values obtained for the 4A zeolite (66.04 m^2^/g; 0.1489 cm^3^/g; 9.02 nm), which suggests that both materials possess comparable secondary porosity not directly related to the crystalline zeolitic structure (probably spaces between crystallites or porosity associated with the use of a binder and secondary pore formation). The V_total_ determined by the single-point method at p/p_0_ = 0.90 is only 0.0658 cm^3^/g (desorption), which is the lowest value in the studied series.

### 3.3. Radium Removal—Saline Mine Water

**Figure 4:**
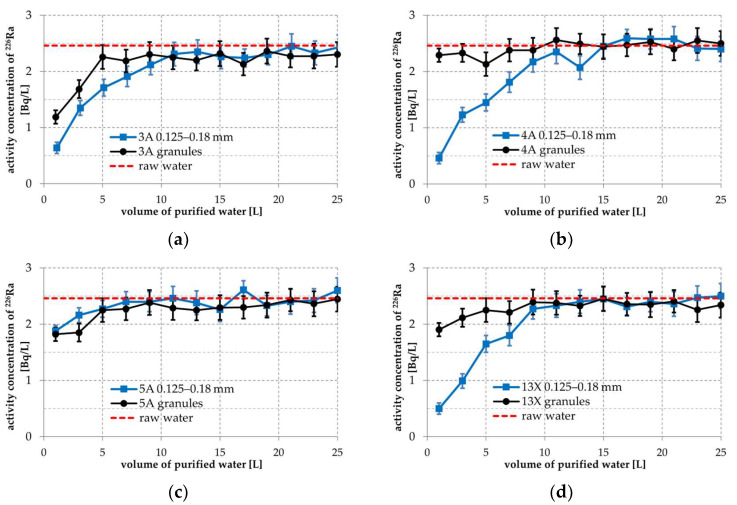
Activity concentration of ^226^Ra versus volume of purified water for zeolites (**a**) 3A, (**b**) 4A, (**c**) 5A, and (**d**) 13X in powder (0.125–0.180 mm) and granular forms.

A series of eight sequential batch experiments for removing radium from collected mine water were carried out, in which four types of zeolites (3A, 4A, 5A and 13X) were tested in two physical forms: fine powder (0.125–0.180 mm fraction) and granules (1.7–2.4 mm diameter); the results are presented in Figure 4. The experiments were first performed with the granular form, which is better suited for water purification in fixed-bed column systems and was therefore initially considered for practical application. However, very low purification efficiencies were obtained. For zeolites 3A (Figure 4a), 5A (Figure 4c) and 13X (Figure 4d), the activity concentration of ^226^Ra in the effluent after treating 5 L of water was within the measurement uncertainty, equal to that in the raw water. In the case of zeolite 4A (Figure 4b), virtually no removal of ^226^Ra was observed at any stage of the experiment. Therefore, it was hypothesized that the granular form might hinder the access of Ra^2+^ ions to the zeolite channels and thus limit the exchange of sodium ions with radium ions. For this reason, the study was extended to include experiments with ground zeolites. After a sieve analysis, the 0.125–0.180 mm fraction was selected. The greatest improvement after grinding was observed for zeolites 4A and 13X, which exhibited noticeable sorption up to approximately 9 L of treated water. Moreover, the removal efficiency (Equation (1)) for the first liter of purified water increased from approximately 5% to 81% for 4A and from 22% to 80% for 13X. For zeolite 3A, the improvement was much smaller (from 52% to 74%), whereas for zeolite 5A, the difference between the granular and fine-powder forms was negligible within the experimental uncertainty.

**Figure 5:**
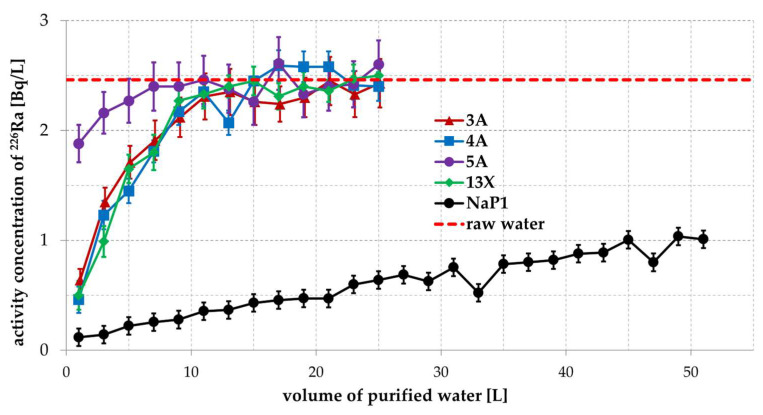
Activity concentration of ^226^Ra versus volume of purified water for zeolites 3A, 4A, 5A,13X and NaP1 in powder form (0.125–0.180 mm).

Because NaP1 was available only as a fine powder, a sieve analysis was performed to separate the 0.125–0.180 mm fraction, so that the purification efficiency of NaP1 could be compared with that of zeolites 3A, 4A, 5A, and 13X. A sequential batch test was conducted using the same radium-bearing mine water as for the aforementioned zeolites. The results of water purification using zeolites 3A, 4A, 5A, 13X, and NaP1, all in the 0.125–0.180 mm fraction, are presented in Figure 5.

In the sequential batch tests conducted for zeolites 3A, 4A and 13X, a distinct increase in the ^226^Ra activity in the treated water was observed with an increasing cumulative treated water volume. These zeolites exhibited very similar radium removal performances. Initially (1–7 L), they markedly reduced the ^226^Ra activity concentration, most effectively for the first liter (down to about 0.5 Bq/L) and for 3 L (to about 1.25 Bq/L). However, after the treatment of 9–11 L of water, the activity concentration in the treated water reached values comparable to those in the raw water. This behavior indicates a gradual exhaustion of the sorption capacity of these materials.

Zeolite 5A showed the weakest performance throughout the experiment; the ^226^Ra activity concentration in the treated water was already approximately 2 Bq/L after the first liter, and became equal to the activity in the raw water after the treatment of only 7 L. This result indicates the low selectivity of zeolite 5A towards radium in the investigated water matrix.

The best results were obtained for NaP1. For this zeolite, the ^226^Ra activity concentration in the treated water remained low over the entire range of treated volumes, and no sharp breakthrough of efficiency was observed. The ^226^Ra activity remained between 0.1 and 0.6 Bq/L for up to 25 L of treated water and was still only about 1 Bq/L after 50 L. An NaP1 sample with a mass of 5.0 g was used to treat 55 L of water, adsorbing 104 Bq of ^226^Ra and 120 Bq of ^228^Ra. These results confirm the high sorption capacity and greater selectivity of NaP1 toward Ra^2+^ ions compared with the other zeolites tested. The superior performance of NaP1 in real saline mine water is consistent with previous studies indicating that low-silica zeolites with high cation exchange capacities exhibit enhanced affinity toward alkaline earth metals and radionuclides [38,74]. The **GIS** framework of NaP1 contains a high density of negatively charged sites associated with the low Si/Al ratio, which promotes divalent cation exchange [75]. Furthermore, fly ash-derived NaP1 materials have previously demonstrated high ion exchange efficiency toward metal ions due to their accessible external surface and heterogeneous pore structure [48,51].

### 3.4. Barium Removal—Synthetic Water

**Figure 6:**
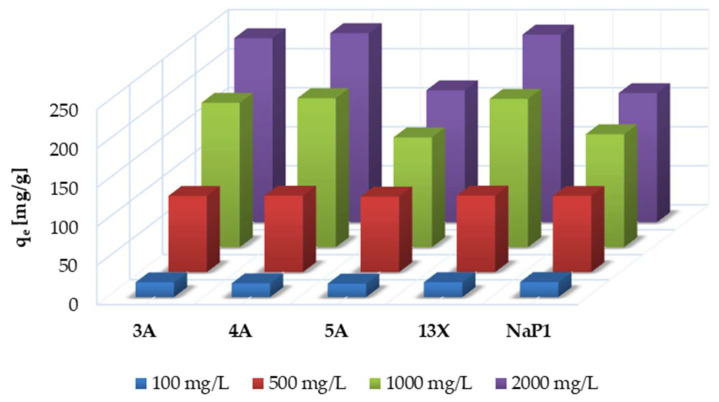
Equilibrium adsorption capacity (q_e_) for 3A, 4A, 5A, 13X and NaP1 zeolites and 100, 500, 1000 and 2000 mg/L barium concentrations.

The adsorption of Ba^2+^ onto zeolites 3A, 4A, 5A, and 13X (in granular form) and NaP1 (in fine-powder form) was studied as a function of initial concentration at 21 °C, using barium concentrations of 100, 500, 1000, and 2000 mg/L, while keeping all other parameters constant. The equilibrium adsorption capacity (qₑ) was calculated according to Equation (2). The results are presented in Table 5 and Figure 6.

For all zeolites, a distinct increase in equilibrium adsorption capacity was observed with an increasing initial barium ion concentration. At initial concentrations of 100 and 500 mg/L, the q_e_ were very similar for all investigated zeolites, close to 20 mg/g and 100 mg/g, respectively, indicating the near-complete removal of barium from the solution under these conditions. The near-complete removal observed at lower Ba^2+^ concentrations indicates that the number of available exchange sites greatly exceeded the amount of dissolved barium under these conditions. Differences between the zeolites became apparent at higher initial barium concentrations. Similar trends have been reported for synthetic and natural zeolites, where the decrease in the percentage removal efficiency with increasing metal loading resulted from the progressive occupation of ion exchange sites in the zeolite structure [76,77]. Zeolite 4A achieved the highest capacities (189.7 and 239.9 mg/g at 1000 and 2000 mg/L), closely followed by 13X (188.8; 238.0 mg/g) and 3A (183.7; 233.4 mg/g). Meanwhile, 5A and NaP1 exhibited mutually similar but lower values (139.5; 166.7 mg/g and 142.9; 166.1 mg/g, respectively).

### 3.5. Zeolite Performance—Comparison and Discussion

The investigated zeolites represent three distinct framework types (**LTA**, **FAU**, and **GIS**), characterized by markedly different pore systems and channel geometries. The ion exchange performance of zeolites is strongly governed by their framework topology, pore size, and the accessibility of exchange sites, which differed significantly between the **LTA**, **FAU**, and **GIS** structures [78]. The **LTA** zeolites 3A and 4A exhibited the highest sorption efficiency for barium, in contrast to zeolite 5A, which shared the same framework structure. In zeolite 5A, calcium acted as the primary exchangeable cation, distinguishing it from the other **LTA**-type zeolites tested in this study. Owing to its double positive charge, calcium was more strongly bound to the framework and more difficult to exchange than the monovalent sodium and potassium ions present in zeolites 3A and 4A [79]. Similar observations were reported by Jurado-Vargas et al., who demonstrated substantially lower exchange efficiencies for Ca-containing zeolites compared with the Na forms [80]. Additionally, the higher hydration energy of divalent Ca^2+^ compared to monovalent cations may have further hindered the ion exchange kinetics. At elevated Ba^2+^ concentrations, zeolite 4A exhibited the highest adsorption capacity, reaching values comparable to those obtained for zeolite 13X, despite the considerably lower S_BET_ and S_Langmuir_ parameters listed in Table 3. Nevertheless, the interpretation of textural properties determined by low-temperature nitrogen adsorption for zeolites 3A and 4A should be approached with caution due to the inherent methodological limitations. These zeolites possess effective pore window diameters of approximately 0.3 and 0.4 nm, respectively, which are similar to the kinetic diameter of nitrogen molecules (~0.364 nm). As a result, nitrogen diffusion into the microporous network was significantly hindered, leading to limited accessibility of the internal surface area to the adsorbate. Consequently, as mentioned, all textural parameters derived from the N_2_ adsorption measurements for the 3A and 4A samples may be substantially underestimated [58]. Zeolite 13X also exhibited an adsorption capacity comparable to that of zeolites 3A and 4A. The good sorption properties of zeolite 13X for Ba were also reported by Sherry [81]. He concluded that at equilibrium, Ba could exchange all Na ions located in one of the two independent but interconnecting three-dimensional cavities, with pores of 9 Å free diameters. NaP1 showed an adsorption capacity for barium very similar to that of zeolite 5A; however, the significantly lower adsorption capacity results obtained for NaP1 compared to zeolites 3A, 4A, and 13X can be explained by the lower content of the zeolite phase in the sample (48% compared to approximately 80%).

The results of the barium sorption experiments performed using synthetic water closely reflect the radium sorption results obtained using real mine water. Both experiments revealed a similar ranking of the tested materials. The lowest removal efficiencies were recorded for zeolite 5A. Zeolites 3A, 4A, and 13X showed similar capabilities for removing radium from water, reaching saturation after the treatment of 9–11 L, when the activity concentration in the treated water reached values comparable to those in the raw water. The best performance, superior to all other materials, was achieved by NaP1. From these experiments, it can be concluded that the selectivity follows the order Ra > Ba for NaP1. The presence of barium ions in the mine water certainly had a negative impact on the potential for radium sorption, as indicated by the experiments performed for barium. Furthermore, it is suspected that the other ions present in the treated water at very high concentrations, that is, strontium, magnesium, and calcium, likely exerted a significant influence. The collected water sample exhibited a very high electrical conductivity of 100,000 µS/cm, and the total dissolved solids were 34.0 ± 3.9 g/L. Such a high ionic strength and the presence of competing cations (Na^+^, Ca^2+^, Mg^2+^) are known to significantly reduce the efficiency of ion exchange processes in zeolites due to the competition for exchange sites and charge screening effects [82]. Although ion exchange is widely recognized as the primary mechanism for ions uptake on zeolites in water [83], direct quantification of the released Na^+^ and Ca^2+^ ions was not feasible in this study. This was due to the extremely high background concentrations of these ions in the real wastewater matrix, shown in Table 2, which masked any minor concentration changes resulting from the exchange process. Despite containing the lowest percentage of the zeolite phase (48%) as determined by the XRD analysis, the NaP1 sample exhibited the highest radium removal efficiency. The other components identified in this sample, namely quartz, mullite, feldspar, calcite, and an amorphous phase, originated from the fly ash used as the precursor material for the NaP1 synthesis. This is a common characteristic of zeolites synthesized from fly ash and is well documented in the literature [84,85,86]. To directly assess the contribution of these non-zeolitic components, a control experiment was conducted using the raw, unconverted fly ash for radium removal. The results showed that the raw fly ash had negligible purification capabilities, with removal efficiencies oscillating around 0%, within the limits of experimental uncertainty. This finding strongly suggests that the adsorption performance of NaP1 is attributable to the synthesized zeolite phase, not the residual precursor materials. This conclusion is further supported by the known properties of the main non-zeolitic constituents. Quartz is recognized as a poor sorbent due to its relatively smooth surface, limited number of active sites, and low adsorption capacity [87]. Furthermore, the strong hydration shells of metal ions in aqueous solutions significantly hinder their adsorption onto the quartz surface [88]. Similarly, unmodified mullite demonstrates very low efficiency for heavy metal removal and typically requires surface functionalization, for example with amine groups (-NH_2_), to become an effective adsorbent [89]. Therefore, the residual quartz and mullite in the NaP1 sample were not expected to contribute significantly to radium adsorption. Feldspars, as framework aluminosilicates with tightly locked structures, lack the open porosity and ion exchange capacity necessary for rapid hydration and ion trapping at room temperature [90]. The unreacted amorphous aluminosilicate glass phase, although structurally disordered, lacks the defined cage structures and high cation exchange capacity characteristic of the newly formed zeolite [91].

Therefore, adsorption experiments conducted in simplified laboratory solutions (especially synthetic water with only one contaminant) may overestimate the performance of certain materials when applied to real water. It has been reported that adsorption capacities determined in idealized laboratory systems are often significantly overestimated compared to those using real water due to the presence of competing ions and complex chemical matrices [74,82]. The results obtained using real mine water clearly demonstrate that the matrix effects critically influence the radium removal efficiency, emphasizing the necessity of testing sorbents under realistic environmental conditions.

The experiments on radium removal using zeolites in the form of granules and fine powder (obtained by grinding and sieve analysis) revealed the influence of sorbent morphology on the purification efficiency. The fine crystalline form was characterized by an increase in the radium removal efficiency, which was particularly evident for zeolites 4A and 13X. The change in the purification efficiency for zeolite 5A was negligible and remained within the limits of measurement uncertainty, whereas for zeolite 3A, it improved by approximately 15–20%. The above results suggest that the diffusion limitations within the granules may have been responsible for the lower radium removal efficiency of zeolites 4A and 13X, as crushing them markedly improved their performance. The results further indicate that mass transfer resistance through the binder matrix in the zeolite granules may have also played a significant role. However, the barium removal results show that the granular form allowed the barium ions to penetrate the interior of the granules and undergo sorption. This is a positive finding because the granular form can also be used to remove ions from contaminated water. This is particularly significant for the application of fixed-bed column construction for water treatment, where the use of a fine crystalline form may encounter numerous problems and consequently prevent the application of this technology [52].

The impact of the original material’s particle size on the exchange rate has been experimentally confirmed in multiple studies involving the removal of metals from water using various zeolite materials. There are studies reporting that a decrease in grain size of zeolites leads to an increase in adsorption capacity of heavy metals. Inglezakis and Grigoropoulou [74] investigated the effects of particle size of natural and modified clinoptilolite on the removal efficiency of heavy metals lead, cooper, iron, and chromium in aqueous solutions. They reported that reducing the particle size from 1.4–1.7 to 0.8–1 mm nearly doubled the total volume of treated solution, at which the breakthrough point was reached. Moreover, the use of clay binders, such as kaolin or attapulgite, in industrial zeolite granulation can significantly affect the surface chemistry and ion exchange behavior [72]. Samolej and Franus [52] also reported that the difference between the grain size of NaP1 in fine crystalline form with fractions < 0.032 and 0.125–0.180 mm in a fixed-bed experiment was as high as 30% and increased with the amount of treated water. Holub et al. reported that decreasing the particle size improves the adsorption and ion exchange performance of the natural zeolite clinoptilolite in copper removal experiments [92].

The literature review revealed that no studies have been conducted under conditions and parameters that would allow for a direct comparison with the results of this research. There are no guidelines for metal sorption experiments on zeolites (or other sorption materials) that specify, for example, the ratio of the volume of a solution with contamination to the sorbent, contaminant concentration values in the solution, solution pH, or temperature. Creating such a set of procedures would enable the comparison of results. The research most similar in scope to the discussion in this paper was presented by Jurado-Vargas et al. [80]. They studied the sorption of barium and radium and mixtures of both elements for zeolites 3A and 5A. The ratio of zeolite to solution was 10 g to 1 L, the pH was 3, the barium concentration was 0.0016 meq of Ba/mL and 0.0144 meq of Ba/mL, corresponding with 110 mg/L and 1000 mg/L of the barium concentration, respectively. The percentage sorption of Ba in zeolite 3A at 110 mg/L was reported as 100 ± 1% and for 5A it was 52 ± 5%; for zeolite 3A at 1000 mg/L it was 55 ± 4% and for 5A it was 23 ± 2%.

The barium sorption results presented in this article differ slightly from those mentioned above. For zeolites 3A and 5A at 100 mg/L, the sorption efficiencies were 95% and 87%, respectively. The sorption efficiencies for zeolites 3A and 5A at 1000 mg/L were 92% and 70%, respectively. In the case of radium sorption in the presence of barium, the radium removal efficiencies were also higher for zeolite 3A than for zeolite 5A, analogous to the results presented in the graphs in Figure 4 and Figure 5. The discrepancies may have resulted from different experimental conditions, such as pH; a different zeolite-to-water ratio; and possible differences in the zeolite phase content in the zeolite sample. However, the most important conclusion from this comparison, which is visible in both the barium and radium sorption studies, seems to be consistent. Zeolite 5A exhibits significantly worse properties for removing barium and radium from water than zeolite 3A. Zeolite 5A is the only zeolite among the tested zeolites that contains calcium ions as the ions balancing the Si-Al network charge. Jurado-Vargas et al. [80] observed that calcium ions were exchanged in the smallest percentage compared to sodium, which is also present in zeolite 5A. Calcium is probably more strongly bound to the zeolite network than sodium, primarily due to its double charge and, thus, removing calcium from zeolite is more difficult than removing sodium. The obtained results confirm this.

In column experiments, Vaaramaa et al. [93] compared the application of zeolite 4A with weak and strong cation exchange resins for the removal of radium from real groundwater sampled from a drilled well. The low efficiency of zeolite 4A was reflected in its poor decontamination factor (DF = C_in_/C_out_) values, which ranged from only three to five. These findings are in agreement with the results presented in this study, confirming the low selectivity of zeolite 4A for radium.

## 4. Conclusions

This study evaluated radium removal from real saline mine water and barium removal from synthetic water using five synthetic zeolites (NaP1, 3A, 4A, 5A, and 13X) in different physical forms. The main contribution of this work is its comparative evaluation of multiple zeolites under both ideal (synthetic water) and realistic (saline mine water) conditions.

With respect to radium removal from the mine water experiments, the key finding is that zeolite NaP1 achieved the highest radium uptake: its effluent ^226^Ra activity remained very low even after treating ~50 L of water, whereas zeolites 3A, 4A, and 13X significantly reduced ^226^Ra only in the first few liters (becoming exhausted after ~9–11 L). Zeolite 5A performed poorly for radium, with its effluent activity concentration reaching raw water levels after only ~7 L. These results highlight NaP1’s strong potential as a highly selective sorbent for Ra^2+^ in complex saline waters. The high efficiency of NaP1 zeolite at removing radium from real mine water does not stem from its absolute ion exchange capacity, pure zeolite phase content, or best textural parameters. Instead, it is governed by its unique selectivity for Ra^2+^. In a complex brine environment characterized by massive concentrations of competing background ions (e.g., 27,000 mg/L Na^+^, 1900 mg/L Ca^2+^, 2100 mg/L Mg^2+^), the highly capacitive but non-selective-for-Ra^2+^ commercial zeolites (4A, 13X) are rapidly exhausted due to matrix complexity.

In the batch tests for barium removal, all the zeolites removed > 95% of Ba^2+^ at low concentrations (100 mg/L). At higher Ba^2+^ loadings (2000 mg/L), zeolites 4A, 13X, and 3A showed the highest adsorption capacities (~233.4–239.9 mg/g), whereas 5A and NaP1 were lower (~166.1–166.7 mg/g). Zeolites 4A, 13X and 3A exhibited the highest capacities in these single-contaminant tests. When removing barium from single-contaminant synthetic solutions, ionic competition is absent. Under these conditions, the textural parameters become one of the key factors. This aligns with the instrumental findings, explaining why commercial zeolites 4A and 13X, possessing the highest structural purity, best textural parameters in the case of 13X, and the largest reserves of exchangeable monovalent sodium cations, yielded the best barium uptake. Simultaneously, the poor performance of zeolite 5A across all tested scenarios is consistent with the lower exchangeability of Ca^2+^ compensating cations, which strongly hinders the ion exchange process. The difference between the granular and powdered forms most likely reflects the mass transfer limitations associated with granulation and binder effects rather than a change in the adsorption mechanism.

The study also emphasizes that laboratory tests in idealized solutions can overestimate performance in real waters. Sorbents must be evaluated in realistic matrices, since natural waters have complex chemistries that can drastically affect sorption efficiency. Another limitation is that the experiments were laboratory-scale; future work should validate NaP1’s performance under field conditions or continuous flow operations to ensure generality. Among the tested materials and under the studied conditions, no better candidate for radium removal from water was identified than the zeolite NaP1. Overall, NaP1 emerges as a strong candidate for practical radium remediation, and the comparative approach provides guidance on choosing and customizing zeolites for different contaminants and water conditions.

## Figures and Tables

**Table 1:** Technical characteristics of commercial zeolite samples (3A, 4A, 5A, and 13X) provided by the manufacturer.

Parameter	3A	4A	5A	13X
shape	sphere	sphere	sphere	sphere
diameter	8–12 mesh	8–12 mesh	8–12 mesh	8–12 mesh
bulk density	≥0.7 g/mL	≥0.7 g/mL	≥0.7 g/mL	≥0.68 g/mL
crushing strength	≥35 N/piece	≥35 N/piece	≥35 N/piece	≥30 N/piece
static H_2_O adsorption	≥21%	≥21.5%	≥21%	≥25%
water content	≤1.5%	≤1.5%	≤1.5%	≤1.5%

**Table 2:** Chemical parameters of collected mine water used in experiments before its treatment with zeolites.

Parameter		Parameter	
pH	7.2	K^+^	470 ± 50 mg/L
Conductivity	100,000 ± 10,000 µS/cm	Ba^2+^	0.2 ± 0.02 mg/L
Total hardness	13,000 ± 120 mg/L CaCO_3_	Sr^2+^	35.0 ± 4.0 mg/L
Ca^2+^	1900 ± 200 mg/L	SO_4_^2−^	2800 ± 300 mg/L
Mg^2+^	2100 ± 200 mg/L	Fe^2+^	0.17 ± 0.01 mg/L
Na^+^	27,000 ± 2700 mg/L	Mn^2+^	1.5 ± 0.2 mg/L

**Table 3:** Elemental composition of zeolites in fine-powder and granular forms.

Zeolite	Form	Si	Al	Na	K	Ca	Mg	Ti	Fe	Si:Al
		**At%**								**-**
3A	fine powder	39.1	32.6	6.9	19.1	0.4	1.1	0.1	0.8	1.20
3A	granules	41.6	31.3	9.2	15.9	<LOD *	2.1	<LOD *	<LOD *	1.33
4A	fine powder	42.1	33.7	21.0	2.2	0.1	0.8	<LOD *	0.2	1.25
4A	granules	44.8	29.4	18.8	2.9	<LOD *	4.1	<LOD *	<LOD *	1.53
5A	fine powder	41.8	36.6	6.9	0.1	11.0	2.2	0.4	1.1	1.14
5A	granules	47.9	32.4	6.2	0.0	10.6	2.9	0.0	0.0	1.48
13X	fine powder	48.0	29.9	15.1	0.5	0.7	2.4	0.2	3.1	1.61
13X	granules	46.9	28.3	21.4	<LOD *	<LOD *	3.5	<LOD *	<LOD *	1.65
NaP1	fine powder	43.4	34.6	18.6	<LOD *	1.6	0.5	0.5	0.9	1.25

* LOD—limit of detection.

**Table 4:** Textural parameters of zeolites (S_BET_—BET surface area; S_Langmuir_—Langmuir surface area; S_mic_—t-plot micropore area; S_ext_—t-plot external surface area; V_total_—total pore volume; V_mic_—t-plot micropore volume).

	3A	4A	5A	NaP1	13X
S_BET_ (m^2^/g)	45.2	64.9	522.7	50.1	631.9
S_Langmuir_ (m^2^/g)	289.2	272.6	630.8	68.1	724.1
S_mic_ t-plot (m^2^/g)	-	-	420.3	1.121	676.8
S_ext_ t-plot (m^2^/g)	67.0	76.9	102.4	49.0	47.3
V_mic_ t-plot (cm^3^/g)	-	-	0.1619	0.0013	0.2203
V_total_ (cm^3^/g)	0.0658	0.0839	0.2619	0.2190	0.2730

**Table 5:** Equilibrium adsorption capacity (q_e_) for zeolites 3A, 4A, 5A, 13X and NaP1, and initial concentration of barium.

Initial Ba^2+^ Concentration	3A	4A	5A	13X	NaP1
			q_e_ (mg/g)		
100 mg/L	18.83 ± 0.35	17.48 ± 0.36	17.18 ± 0.36	18.89 ± 0.35	19.05 ± 0.35
500 mg/L	94.22 ± 1.78	95.31 ± 1.76	92.50 ± 1.77	95.54 ± 1.76	95.06 ± 1.76
1000 mg/L	183.7 ± 3.6	189.7 ± 3.5	139.5 ± 3.8	188.8 ± 3.5	142.9 ± 3.8
2000 mg/L	233.4 ± 8.0	239.9 ± 7.9	166.7 ± 8.4	238.0 ± 8.1	166.1 ± 8.4

## Data Availability

The original contributions presented in this study are included in the article. Further inquiries can be directed to the corresponding author.
